# Skin-testing protocol in diagnosing of the IgE-mediated peanut allergy

**DOI:** 10.1186/2045-7022-5-S3-P160

**Published:** 2015-03-30

**Authors:** Lotfollah Behroo, Farideh Shishehbor, Mehri Ghafourian

**Affiliations:** 1 Nutrition and Metabolic Diseases Research Center, Ahvaz Jundishapur University of Medical Sciences, Ahvaz, Iran; 2 Department of Immunology, Fertility, Infertility and Perinatology Research Center, Ahvaz Jundishapur, Ahvaz, Iran

## Background

In the event that a food-allergen was presumed as a likely accused of signs/symptoms, after that, the substantiation of major associations and finally, identifying the incriminated food(s) need to be warranted.

As for verifying of an IgE-mediated food allergy, different specific mensurations should be accomplished in order to include/exclude the suspected food(s). One protocol for meticulous interpretation −concerning the production of specific IgE-antibodies− is the accepted/endorsed prick-puncture skin-testing.

Of note, Skin-Prick-Tests (SPTs) are referred to as authentic predicting-value in cases where they become negative (over 95 %).

## Methods

Methodically, Ventral surfaces of wistar rats were utilised for *intra-dermal* skin-testing with Crude Peanut Extract (CPE). So primarily, 5-min before the test, 100 µL of Evan's blue dye was administered into the tail-vein to aid visualising the wheal reaction.

Subsequently, 66 µL of the filter-sterilised CPE was injected *intra-dermally* into the already shaved abdominal skin. (Negative counterparts were placed, too).

A positive test-response was defined as a wheal-reaction emerging as a blue bump sizing diametrically, greater than 5 mm.

## Results

Predictably, abdominal aspects of the examined wistars, merely in positive group, revealed a wheal-reaction as a blue circle close to 1-cm in diameter, while recording −20-min subsequent to *id* peanut-challenge.

## Conclusions

Putting together, although animal models carry actually, advantageous responsibilities in inspecting the food-allergy patho-mechanisms, and so forth, Nonetheless, a variety of validating researches remain still to be performed. On this concern, any required, efficacious and qualified instructions, as well as, reliable conducts might be accurately familiarised for imitating the complications of interest, and eventually, recruiting the recently obtained information in forthcoming human-subject experiments.

